# 3,5-Dihydr­oxy-2-methyl-4*H*-pyran-4-one

**DOI:** 10.1107/S1600536808010957

**Published:** 2008-05-10

**Authors:** Cheng-Ming Dong, Shou-Cheng Pu, Wen-Yuan Gao

**Affiliations:** a Department of Pharmaceutical Science, Henan College of Traditional Chinese Medicine, Zhengzhou 450008, People’s Republic of China; b School of Pharmaceutical Science & Technology, Tianjin University, Tianjin 300072, People’s Republic of China

## Abstract

In the title compound, C_6_H_6_O_4_, inter- and intra­molecular hydrogen bonds are observed which help to establish the crystal structure. There are weak π–π interactions between pyran rings separated by 3.5692 (9) Å.

## Related literature

For general background, see: Shinoda *et al.* (2004 ▶). For related structures, see: Yao *et al.* (2005 ▶); Gibbons *et al.* (2000 ▶).
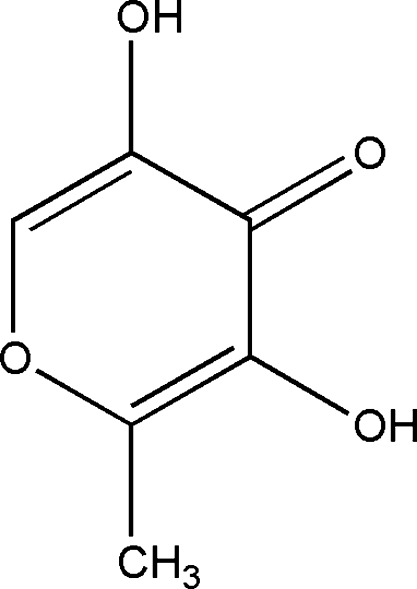


## Experimental

### 

#### Crystal data

C_6_H_6_O_4_
*M* *_r_* = 142.11
Monoclinic, 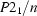

*a* = 6.9400 (14) Å
*b* = 6.0648 (12) Å
*c* = 14.008 (3) Å
β = 92.77 (3)°
*V* = 588.9 (2) Å^3^
*Z* = 4
Mo *K*α radiation
μ = 0.14 mm^−1^
*T* = 113 (2) K
0.14 × 0.12 × 0.10 mm

#### Data collection

Rigaku Saturn diffractometer
Absorption correction: multi-scan (*CrystalClear*; Rigaku/MSC, 2005 ▶) *T*_min_ = 0.981, *T*_max_ = 0.986
3970 measured reflections
1381 independent reflections
1166 reflections with *I* > 2σ(*I*)
*R*_int_ = 0.025

#### Refinement

*R*[*F* ^2^ > 2σ(*F* ^2^)] = 0.032
*wR*(*F* ^2^) = 0.096
*S* = 1.10
1381 reflections
115 parameters
All H-ataom parameters refined
Δρ_max_ = 0.37 e Å^−3^
Δρ_min_ = −0.24 e Å^−3^

### 

Data collection: *CrystalClear* (Rigaku/MSC, 2005 ▶); cell refinement: *CrystalClear*; data reduction: *CrystalClear*; program(s) used to solve structure: *SHELXS97* (Sheldrick, 2008 ▶); program(s) used to refine structure: *SHELXL97* (Sheldrick, 2008 ▶); molecular graphics: *SHELXTL* (Sheldrick, 2008 ▶); software used to prepare material for publication: *SHELXTL*.

## Supplementary Material

Crystal structure: contains datablocks I, global. DOI: 10.1107/S1600536808010957/pv2074sup1.cif

Structure factors: contains datablocks I. DOI: 10.1107/S1600536808010957/pv2074Isup2.hkl

Additional supplementary materials:  crystallographic information; 3D view; checkCIF report

## Figures and Tables

**Table 1:** Hydrogen-bond geometry (Å, °)

*D*—H⋯*A*	*D*—H	H⋯*A*	*D*⋯*A*	*D*—H⋯*A*
O4—H6⋯O3^i^	0.838 (18)	1.89 (2)	2.6902 (12)	159.6 (13)
O2—H5⋯O3^ii^	0.94 (2)	1.75 (2)	2.6596 (12)	162.6 (17)
O4—H6⋯O3	0.838 (18)	2.44 (2)	2.7820 (12)	105.4 (10)
C1—H3⋯O4	1.005 (15)	2.537 (14)	2.8957 (15)	100.5 (9)
C6—H4⋯O2^iii^	0.936 (14)	2.412 (13)	3.3354 (14)	169.4 (12)

Symmetry codes: (i) 
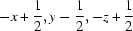
; (ii) 
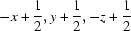
; (iii) 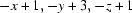.

**Fig. 1.**
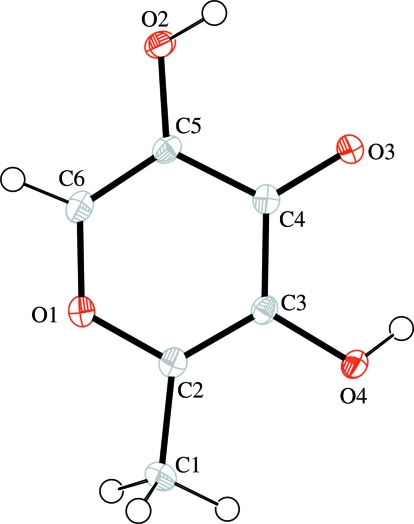
A view of the molecule of (I). Displacement ellipsoids are drawn at the 30% probability level and H atoms are shown as small spheres of arbitrary radii.

## supplementary crystallographic information

### Comment

The title compound, 3,5-dihydroxy-2-methyl-pyran-4-one, (I) was identified as a decomposition product in the stored solution of orange juice (Shinoda, *et al.*, 2004). We report here the crystal structure of the title compound (Fig. 1) which was isolated from Hydrocotyle sibthorpoioides Lam. The structure of (I) is stabilized by two strong intermolecular hydrogen bonds of the type O—H···O and a weak intermolecular interaction of the type C—H···O. Intramolecular interactions are also observed which result in five membered rings; details are given in Table 1. There is indication of π-π interactions between the pyran rings lying about inversion centers with minimum separation of 3.5692 (9) Å. The crystal structures of 2-hydroxymethyl analogue (Yao *et al.*, 2005) and 5-hydroxy-3-methoxy-pyran-4-one (Gibbons *et al.*, 2000) have been reported.

### Experimental

Dried powder of Hydrocotyle sibthorpoioides Lam was exacted with EtOH and the extract was concentrated *in vacuo*. The residue was subjected to silical-gel coloumn chromatography. Elution with chloroform-methanol (95:5 *v*/*v*) yielded the title compound. Crystals suitable for XRD study were grwon from a solution of methanol at room temperature by slow evaporation.

### Refinement

All H atoms were located from difference map and allowed to refine freely.

### Crystal data

**Table:**

C_6_H_6_O_4_	*F*_000_ = 296
*M**_r_* = 142.11	*D*_x_ = 1.603 Mg m^−^^3^
Monoclinic, *P*2_1_/*n*	Mo *K*α radiation λ = 0.71073 Å
Hall symbol: -P 2yn	Cell parameters from 1620 reflections
*a* = 6.9400 (14) Å	θ = 1.5–27.9º
*b* = 6.0648 (12) Å	µ = 0.14 mm^−^^1^
*c* = 14.008 (3) Å	*T* = 113 (2) K
β = 92.77 (3)º	Block, colorless
*V* = 588.9 (2) Å^3^	0.14 × 0.12 × 0.10 mm
*Z* = 4	

### Data collection

**Table:**

Rigaku Saturn diffractometer	1381 independent reflections
Radiation source: rotating anode	1166 reflections with *I* > 2σ(*I*)
Monochromator: confocal	*R*_int_ = 0.025
*T* = 113(2) K	θ_max_ = 27.9º
ω scans	θ_min_ = 2.9º
Absorption correction: multi-scan(CrystalClear; Rigaku/MSC, 2005)	*h* = −9→9
*T*_min_ = 0.981, *T*_max_ = 0.986	*k* = −7→7
3970 measured reflections	*l* = −10→18

### Refinement

**Table:**

Refinement on *F*^2^	Secondary atom site location: difference Fourier map
Least-squares matrix: full	Hydrogen site location: inferred from neighbouring sites
*R*[*F*^2^ > 2σ(*F*^2^)] = 0.032	All H-atom parameters refined
*wR*(*F*^2^) = 0.096	*w* = 1/[σ^2^(*F*_o_^2^) + (0.0654*P*)^2^] where *P* = (*F*_o_^2^ + 2*F*_c_^2^)/3
*S* = 1.11	(Δ/σ)_max_ < 0.001
1381 reflections	Δρ_max_ = 0.37 e Å^−^^3^
115 parameters	Δρ_min_ = −0.24 e Å^−^^3^
Primary atom site location: structure-invariant direct methods	Extinction correction: none

### Special details

**Table:**

Geometry. All e.s.d.'s (except the e.s.d. in the dihedral angle between two l.s. planes) are estimated using the full covariance matrix. The cell e.s.d.'s are taken into account individually in the estimation of e.s.d.'s in distances, angles and torsion angles; correlations between e.s.d.'s in cell parameters are only used when they are defined by crystal symmetry. An approximate (isotropic) treatment of cell e.s.d.'s is used for estimating e.s.d.'s involving l.s. planes.
Refinement. Refinement of *F*^2^ against ALL reflections. The weighted *R*-factor *wR* and goodness of fit *S* are based on *F*^2^, conventional *R*-factors *R* are based on *F*, with *F* set to zero for negative *F*^2^. The threshold expression of *F*^2^ > σ(*F*^2^) is used only for calculating *R*-factors(gt) *etc*. and is not relevant to the choice of reflections for refinement. *R*-factors based on *F*^2^ are statistically about twice as large as those based on *F*, and *R*- factors based on ALL data will be even larger.

### Fractional atomic coordinates and isotropic or equivalent isotropic displacement parameters (Å^2^)

**Table:**

	*x*	*y*	*z*	*U*_iso_*/*U*_eq_	
O3	0.27997 (10)	1.01120 (11)	0.27250 (5)	0.0149 (2)	
O1	0.76427 (10)	1.05517 (12)	0.44524 (5)	0.0157 (2)	
O4	0.56909 (12)	0.69576 (12)	0.26153 (5)	0.0171 (2)	
O2	0.32648 (11)	1.36372 (12)	0.40092 (5)	0.0173 (2)	
C4	0.43152 (15)	1.02588 (15)	0.32615 (7)	0.0124 (2)	
C5	0.46324 (15)	1.20540 (16)	0.39217 (7)	0.0132 (2)	
C3	0.58183 (15)	0.86536 (16)	0.32479 (7)	0.0127 (2)	
C2	0.74196 (15)	0.88197 (16)	0.38483 (7)	0.0139 (2)	
C6	0.62774 (16)	1.21333 (17)	0.44749 (7)	0.0157 (2)	
C1	0.90601 (15)	0.72547 (19)	0.39113 (8)	0.0174 (3)	
H4	0.656 (2)	1.323 (2)	0.4929 (10)	0.021 (3)*	
H3	0.871 (2)	0.582 (2)	0.3585 (10)	0.028 (3)*	
H1	1.017 (2)	0.782 (2)	0.3616 (11)	0.037 (4)*	
H2	0.940 (2)	0.689 (2)	0.4591 (10)	0.025 (3)*	
H5	0.271 (3)	1.393 (3)	0.3397 (14)	0.054 (5)*	
H6	0.454 (3)	0.669 (2)	0.2453 (11)	0.037 (4)*	

### Atomic displacement parameters (Å^2^)

**Table:**

	*U*^11^	*U*^22^	*U*^33^	*U*^12^	*U*^13^	*U*^23^
O3	0.0141 (4)	0.0150 (4)	0.0153 (4)	0.0004 (3)	−0.0022 (3)	−0.0013 (3)
O1	0.0155 (4)	0.0165 (4)	0.0148 (4)	−0.0005 (3)	−0.0020 (3)	−0.0020 (3)
O4	0.0136 (4)	0.0163 (4)	0.0211 (4)	0.0004 (3)	−0.0008 (3)	−0.0080 (3)
O2	0.0233 (4)	0.0142 (4)	0.0142 (4)	0.0058 (3)	−0.0018 (3)	−0.0018 (3)
C4	0.0142 (5)	0.0125 (5)	0.0105 (4)	−0.0022 (4)	0.0016 (4)	0.0017 (3)
C5	0.0177 (5)	0.0106 (5)	0.0116 (4)	0.0007 (4)	0.0021 (4)	0.0008 (3)
C3	0.0138 (5)	0.0118 (5)	0.0128 (5)	−0.0019 (4)	0.0023 (4)	−0.0011 (3)
C2	0.0145 (5)	0.0141 (5)	0.0132 (4)	−0.0017 (4)	0.0024 (4)	−0.0003 (3)
C6	0.0200 (6)	0.0133 (5)	0.0139 (5)	−0.0012 (4)	0.0006 (4)	−0.0020 (4)
C1	0.0126 (5)	0.0200 (6)	0.0195 (5)	0.0013 (4)	0.0001 (4)	−0.0010 (4)

### Geometric parameters (Å, °)

**Table:**

O3—C4	1.2659 (13)	C4—C5	1.4386 (13)
O1—C6	1.3497 (13)	C5—C6	1.3494 (16)
O1—C2	1.3531 (12)	C3—C2	1.3646 (15)
O4—C3	1.3577 (12)	C2—C1	1.4816 (15)
O4—H6	0.838 (18)	C6—H4	0.936 (14)
O2—C5	1.3598 (12)	C1—H3	1.005 (15)
O2—H5	0.94 (2)	C1—H1	0.956 (17)
C4—C3	1.4276 (14)	C1—H2	0.996 (15)
			
C6—O1—C2	120.47 (8)	O1—C2—C3	120.53 (9)
C3—O4—H6	110.7 (11)	O1—C2—C1	113.31 (9)
C5—O2—H5	107.9 (12)	C3—C2—C1	126.15 (9)
O3—C4—C3	122.06 (9)	C5—C6—O1	122.45 (9)
O3—C4—C5	122.13 (9)	C5—C6—H4	124.0 (8)
C3—C4—C5	115.82 (9)	O1—C6—H4	113.5 (8)
C6—C5—O2	119.86 (9)	C2—C1—H3	111.1 (8)
C6—C5—C4	119.68 (10)	C2—C1—H1	112.2 (9)
O2—C5—C4	120.44 (9)	H3—C1—H1	107.1 (13)
O4—C3—C2	118.92 (9)	C2—C1—H2	110.3 (8)
O4—C3—C4	120.04 (9)	H3—C1—H2	106.4 (12)
C2—C3—C4	121.01 (9)	H1—C1—H2	109.5 (13)
			
O3—C4—C5—C6	−179.79 (9)	C6—O1—C2—C1	179.47 (9)
C3—C4—C5—C6	0.12 (14)	O4—C3—C2—O1	176.47 (9)
O3—C4—C5—O2	1.94 (15)	C4—C3—C2—O1	−1.93 (15)
C3—C4—C5—O2	−178.15 (8)	O4—C3—C2—C1	−2.39 (16)
O3—C4—C3—O4	3.12 (15)	C4—C3—C2—C1	179.21 (9)
C5—C4—C3—O4	−176.78 (8)	O2—C5—C6—O1	176.71 (9)
O3—C4—C3—C2	−178.50 (9)	C4—C5—C6—O1	−1.58 (15)
C5—C4—C3—C2	1.59 (14)	C2—O1—C6—C5	1.32 (15)
C6—O1—C2—C3	0.47 (15)		

### Hydrogen-bond geometry (Å, °)

**Table:**

*D*—H···*A*	*D*—H	H···*A*	*D*···*A*	*D*—H···*A*
O4—H6···O3^i^	0.838 (18)	1.89 (2)	2.6902 (12)	159.6 (13)
O2—H5···O3^ii^	0.94 (2)	1.75 (2)	2.6596 (12)	162.6 (17)
O4—H6···O3	0.838 (18)	2.44 (2)	2.7820 (12)	105.4 (10)
C1—H3···O4	1.005 (15)	2.537 (14)	2.8957 (15)	100.5 (9)
C6—H4···O2^iii^	0.936 (14)	2.412 (13)	3.3354 (14)	169.4 (12)

Symmetry codes: (i) −*x*+1/2, *y*−1/2, −*z*+1/2; (ii) −*x*+1/2, *y*+1/2, −*z*+1/2; (iii) −*x*+1, −*y*+3, −*z*+1.
